# The yoga of Rag GTPases: Dynamic structural poses confer amino acid sensing by mTORC1

**DOI:** 10.1016/j.jbc.2021.101103

**Published:** 2021-08-20

**Authors:** Diane C. Fingar

**Affiliations:** Department of Cell and Developmental Biology, University of Michigan Medical School, Ann Arbor, Michigan, USA

**Keywords:** CRD, C-terminal roadblock domain, mTOR, mechanistic target of rapamycin, mTORC1, mechanistic target of rapamycin complex 1, NBD, nucleotide-binding domain, Rheb, Ras homolog enriched in the brain

## Abstract

Heterodimeric Rag GTPases play a critical role in relaying fluctuating levels of cellular amino acids to the sensor mechanistic target of rapamycin complex 1. Important mechanistic questions remain unresolved, however, regarding how guanine nucleotide binding enables Rag GTPases to transition dynamically between distinct yoga-like structural poses that control activation state. Egri and Shen identified a critical interdomain hydrogen bond within RagA and RagC that stabilizes their GDP-bound states. They demonstrate that this long-distance interaction controls Rag structure and function to confer appropriate amino acid sensing by mechanistic target of rapamycin complex 1.

Mechanistic target of rapamycin complex 1 (mTORC1) integrates diverse cellular cues to promote cell growth and proliferation (1, 2). Sufficient levels of nutrients such as amino acids are required for growth factors and hormones (*e.g.*, IGF-1 and insulin) to activate mTORC1 via PI3K, Akt, Ras homolog enriched in the brain (Rheb) (a small GTPase), and tuberous sclerosis complex (a GTPase-activating protein for Rheb) (Fig. 1*A*). mTORC1 signaling in turn drives anabolic (*e.g.*, protein synthesis) and suppresses catabolic (*e.g.*, autophagy) cellular processes. Evolutionarily conserved Rag GTPases play a critical role in amino acid sensing by mTORC1 (3, 4). Despite advances in understanding Rag structure and function, important mechanistic questions remain regarding how dynamic structural states of Rag proteins controlled by guanine nucleotide binding confer amino acid sensing by mTORC1. Egri and Shen used elegant kinetic and cell-based methods to quantitatively dissect dynamic structural elements within Rag subunits that enable mTORC1 to respond to fluctuating levels of amino acids appropriately and rapidly (5).

**Figure 1:**
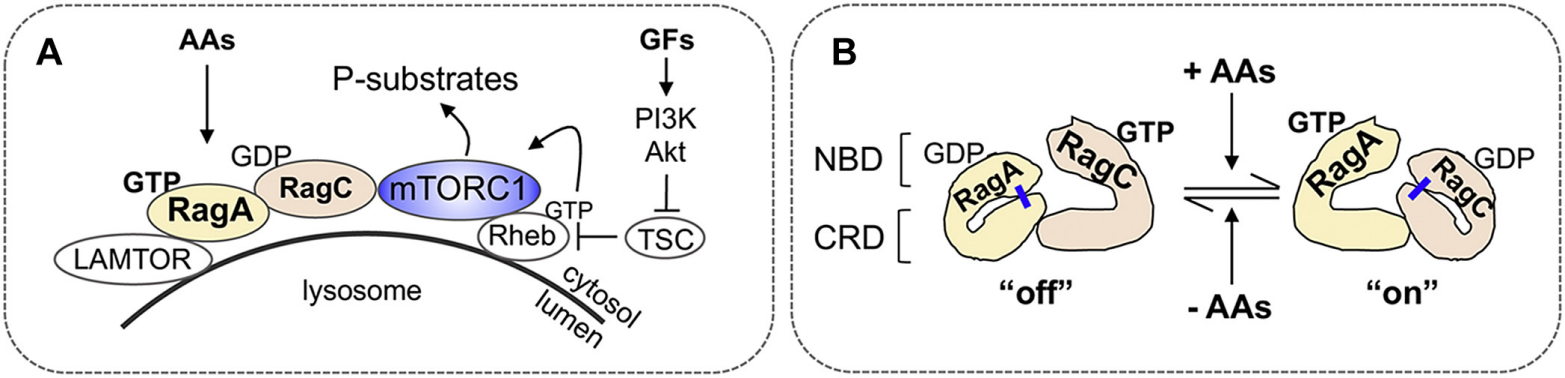
**mTORC1 activation by growth factors (GFs) requires sufficient levels of amino acids (AAs).** GFs and hormones (*e.g.*, IGF-1; insulin) signal through PI3K, Akt, and TSC and activate Rheb through increased GTP loading (*A*). AAs drive Rag heterodimers toward a RagA/B^GTP^–RagC/D^GDP^ “on” state; conversely, AA deprivation induces a switch toward a RagA/B^GDP^–RagC/D^GTP^ “off” state. In the “on” state, Rag heterodimers bind to and recruit mTORC1 to the surface of lysosomes, where Rheb resides. Therefore, AAs and GFs activate mTORC1 cooperatively because of an induced proximity mechanism mediated by Rags and Rheb. A critical hydrogen bond (*blue bar*) between the NBD and CRD of RagA or RagC plays a critical role in maintaining the two stable “on” and “off” states (*B*). CRD, C-terminal roadblock domain; mTORC1, mechanistic target of rapamycin complex 1; NBD, nucleotide-binding domain; Rheb, Ras homolog enriched in the brain; TSC, tuberous sclerosis complex.

Rag proteins function as obligate heterodimers, whereby mammalian RagA or RagB dimerizes with RagC or RagD. Rag proteins localize to lysosomal membranes by tethering to the LAMTOR/Ragulator complex (Fig. 1*A*) (6). In the active RagA/B^GTP^–RagC/D^GDP^ state formed in amino acid–replete conditions, the Rag heterodimer recruits mTORC1 to the lysosomal surface through direct binding (6). Such recruitment enables Rheb to associate with and activate mTORC1 by an induced proximity mechanism (7). Upon amino acid withdrawal, GTP on RagA/B hydrolyzes to GDP, and GTP exchanges for GDP on RagC/D. This inactive RagA/B^GDP^–RagC/D^GTP^ heterodimer releases mTORC1 into the cytosol. Thus, Rags function as dynamic molecular switches that control mTORC1 signaling in accordance with amino acid levels.

Prior work (8) demonstrated that the two GTPase subunits of the Rag heterodimer (RagA/B and RagC/D) communicate with each other. GTP binding to one subunit limits binding of GTP to the other subunit and increases GTP hydrolysis if binding were to occur, and vice versa. Such intersubunit crosstalk prevents dual GTP loading, thus maintaining an opposite guanine nucleotide–loaded state and driving Rag heterodimers into two stable “on” or “off” states. The crystal structure of Rag heterodimers from budding yeast bound to GDP or GTP provided important structural information regarding how guanine nucleotide binding controls Rag architecture (9, 10). An individual Rag subunit consists of a nucleotide-binding domain (NBD) and a C-terminal roadblock domain (CRD) that mediates heterodimerization. In the GDP-bound state, the switch I domain within the NBD forms an alpha helix that orients toward the CRD; in the GTP-bound state, the switch I domain swings upward to the top of the nucleotide-binding pocket, away from the CRD. From the yeast Rag crystal structures (9, 10), Egri and Shen predicted that in the GDP- but not GTP-bound state, the hydroxyl group of Ser266 in the RagC CRB forms hydrogen bonds with Lys84 in the switch I alpha helix of the RagC NBD. As RagA Thr210 is analogous to RagC Ser266, they also predicted that Thr210 in the RagA CRB forms hydrogen bonds with Asn30 in the NBD. In the GTP-bound state, the switch I domain swings up and away from the CRD, preventing formation of these hydrogen bonds (Fig. 1*B*).

Egri and Shen coupled these predictions with elegant quantitative kinetic *in vitro* assays of guanine nucleotide loading and GTP hydrolysis to demonstrate that a critical interdomain interaction in RagA and RagC maintains an opposite nucleotide-loading state in heterodimers and regulates mTORC1 activity (5). They first mutated RagA Thr210 and RagC Ser266 to Ala to abrogate the hydrogen bond and then biochemically purified WT and mutant Rag heterodimers. Ablation of the hydrogen bond had no effect on guanine nucleotide binding. When only one GTP was bound to the heterodimer, rates of GTP hydrolysis were similar on WT and mutant Rag heterodimers. When both Rag subunits of the heterodimer were forced to bind GTP, WT heterodimers displayed an increased rate of GTP hydrolysis compared with those loaded with a single GTP, indicating that the heterodimer actively resolves the dual GTP problem by hydrolyzing GTP on one subunit, consistent with prior work (8). GTP hydrolysis was increased even more for the RagA(T210A)–RagC and RagA–RagC(S266A) mutant heterodimers, suggesting that the mutations mimic a constitutive GTP-loaded conformation, driving faster GTP hydrolysis on the other subunit. In WT heterodimers, preloading the first subunit with GTP increased GTP hydrolysis on the other subunit relative to preloading with GDP. Interestingly, radioactive GTP hydrolysis in mutant heterodimers was strikingly faster than that of the WT when preloaded with either GTP or GDP, indicating that the RagA(T210) and RagC(S266A) mutations shift the heterodimer toward the GTP-loaded conformation. These results suggest that the hydrogen bond stabilizes the GDP-loaded state, and in its absence, Rag proteins tend to adopt a GTP-bound conformation even when bound to GDP, which accelerates GTP hydrolysis on the other subunit.

Egri and Shen also investigated the functional significance of the RagA and RagC hydrogen bond in the control of mTORC1 signaling. Coimmunoprecipitation experiments and analysis of mTORC1 signaling to its well-established substrate S6K1 in intact cells demonstrated that the RagA(T210A)–RagC mutant associated with and activated mTORC1 inappropriately in the absence of amino acids. Upon amino acid stimulation, the RagA–RagC(S266A) mutant displayed reduced mTORC1 binding and failed to activate mTORC1 signaling. These results are consistent with RagA(T210A) mimicking a RagA^GTP^ “on” state and RagC(S266A) mimicking a RagC^GTP^ “off” state. Taken together, these results reveal the functional significance of the RagA and RagC interdomain hydrogen bond, demonstrating that it plays a critical role in regulation of mTORC1 signaling in accordance with amino acid levels.

Mechanistic understanding of Rag heterodimer asanas (*i.e.*, postures and poses) will improve our understanding of the role of mTORC1 in tumorigenesis and metabolism. For example, cancer-associated mutations have been identified in RagC, which increase mTORC1 binding (2). In addition, the physiologic importance of Rag proteins in metabolic control was demonstrated in mice engineered with an active RagA knock-in allele conferring constitutive GTP loading. These mice die perinatally, as they are unable to suppress mTORC1 signaling appropriately upon severance of the placental nutrient supply at birth. These mice fail to suppress energy expenditure, fail to induce autophagy and liberate amino acids as substrates for gluconeogenesis, and consequently fail to upregulate hepatic glucose production, responses essential for survival during fasting, unlike WT neonates (2). Thus, Rag GTPases play critical roles in cell and organismal physiology. Moving forward, deeper mechanistic insight into the yoga of Rag GTPases will improve our understanding of nutrient sensing, how its aberrant regulation contributes to a host of diseases such as cancer, obesity, and type II diabetes, and how its therapeutic targeting could treat these disorders. Namaste.

## Conflict of interest

The authors declare that they have no conflicts of interest with the contents of this article.
